# Correction: A Fast Incremental Gaussian Mixture Model

**DOI:** 10.1371/journal.pone.0141942

**Published:** 2015-10-28

**Authors:** Rafael Coimbra Pinto, Paulo Martins Engel

The Data Availability Statement for this paper is incorrect. The correct Data Availability Statement is: Data are available at Figshare (http://figshare.com/articles/A_Fast_Incremental_Gaussian_Mixture_Model/1552030). The MNIST data set is available at (http://yann.lecun.com/exdb/mnist/) and the CIFAR10 data set is available at (http://www.cs.toronto.edu/~kriz/cifar.html). The software binaries and source code are deposited to GitHub (https://github.com/rafaelcp/figmn).

## References

[1] Pinto RC, Engel PM (2015) A Fast Incremental Gaussian Mixture Model. PLoS ONE 10(10): e0139931 doi: 10.1371/journal.pone.0139931 26444880 10.1371/journal.pone.0139931 PMC4596621

